# Preventing Sexual Violence in Later Life: Examining Pre- and Post- Bystander Training Results

**DOI:** 10.1093/geroni/igaf122.4353

**Published:** 2025-12-31

**Authors:** Michelle Hand

**Affiliations:** George Mason University, Fairfax, Virginia, United States

## Abstract

Pressing needs persist for practitioner trainings on sexual violence (SV) in later life, which bystander programs have been recommended to address. Yet before this research, no known bystander training on SV in later life existed. This study aimed (a) to explore training needs that may exist for practitioners who serve older adults or survivors and their recommendations for refining a bystander training to prevent SV in later life, then (b) to pilot and evaluate the effectiveness of this training. An interview and focus groups were conducted with 16 practitioners who serve older adults or survivors, and with people older than 50, whose recommendations were used to refine the training, which was piloted with 51 practitioners who serve older adults and survivors. Before the training, participants were asked the extent to which they believed SV in later life is a problem, their intentions to help prevent and address it, and a scenario involving SV in later life was presented, after which participants were asked the extent to which they felt obligated to prevent SV in later life, whether they knew where to report, and if their help was required. These questions were repeated post-training. Although modest improvements yet no significant changes were found in beliefs of SV in later life requiring one’s help or knowing where to report, paired samples t-test results demonstrated significant positive changes across all other measures, reflecting increased awareness of SV in later life, intention and obligation to help prevent and address it. Implications will be discussed.

